# UBQLN4 is activated by C/EBPβ and exerts oncogenic effects on colorectal cancer via the Wnt/β-catenin signaling pathway

**DOI:** 10.1038/s41420-021-00795-4

**Published:** 2021-12-20

**Authors:** Xiaolong Tang, Yahang Liang, Guorui Sun, Qingsi He, Hui Qu, Peng Gao

**Affiliations:** 1 Department of General Surgery, Qilu Hospital of Shandong University, Jinan, 250012 China; 2 Department of Pathology, Qilu Hospital of Shandong University, Jinan, 250012 China

**Keywords:** Colorectal cancer, Prognostic markers

## Abstract

Ubiquilin 4 (UBQLN4) is an important member of the ubiquitin-like protein family. An increasing number of studies have shown that UBQLN4 is an important regulator of tumorigenesis. Nevertheless, the biological function and detailed mechanisms of UBQLN4 in colorectal cancer (CRC) development and progression remain unclear. Here, we identified UBQLN4 upregulation in CRC tissues and it is positively associated with CRC size, TNM stage, and lymphatic metastasis. Patients with high UBQLN4 expression had a poor prognosis. Functionally, overexpression of UBQLN4 significantly promoted CRC cell proliferation, migration, and invasion, while UBQLN4 silencing elicited the opposite effect. This result was consistent with the conclusion that UBQLN4 expression correlated positively with the CRC size and lymphatic metastasis. In vivo, UBQLN4 silencing also inhibited tumor growth. Mechanistically, using gene set enrichment analysis (GSEA) and western blot experiments, we identified that UBQLN4 activated the Wnt/β-catenin signaling pathway to upregulate β-catenin and c-Myc expression, thereby promoting CRC proliferation, migration and invasion. A rescue experiment further verified this conclusion. Dual luciferase reporter, real-time quantitative PCR (RT-qPCR), western blot and chromatin immunoprecipitation (ChIP) assays indicated that the transcription factor CCAAT/enhancer-binding protein beta (C/EBPβ) directly bound to the UBQLN4 core promoter region and activated its transcription, upregulating β-catenin and c-Myc expression to promote CRC progression. Thus, our findings suggest that UBQLN4 is a key oncogene in CRC and may be a promising target for the diagnosis and treatment of patients with CRC.

## Introduction

Colorectal cancer (CRC) is the third most common malignant tumor and ranks second in cancer mortality worldwide, just after lung cancer [1, 2]. With the continuous progress in and development of CRC surgical operations, surgical concepts and surgical adjuvant devices, the long-term survival rate of CRC patients has been improved based on surgical operations aided by postoperative standardized chemoradiotherapy and immunotherapy [3–5]. However, most patients with advanced CRC still have a poor prognosis because of the high risk of recurrence and metastasis after surgery. Therefore, studies of the mechanisms leading to the recurrence and metastasis of CRC and active explorations of effective molecular therapeutic targets are crucial.

The development and progression of CRC involves a wide variety of causes, including genetic mutations [6], abnormal gene fusions [7], copy number variations [8], epigenetic changes [9], and other processes. With the increase in the aforementioned mutations in the cells, the intracellular environment is disturbed, leading to cell carcinogenesis. Cells have numerous defense mechanisms against alterations in the intracellular environment, among which the ubiquitin–proteasome system is particularly important. Some studies have shown that the ubiquitin–proteasome system plays a vital role in regulating and maintaining intracellular homeostasis [10]. Abnormal ubiquitin–proteasome degradation pathways induce various diseases, including viral infectious diseases, congenital dysplasia, neurodegenerative diseases, and cancers [11–14].

Ubiquilin 4 (UBQLN4) is an important member of the ubiquitin-like protein family. This protein is a vital regulator of the degradation process mediated by the ubiquitin–proteasome pathway, which activates the proteasome to target misfolded, mislocalized or stacked proteins [15]. More and more studies have shown that UBQLN4 is a vital tumor-associated gene. Shiloh et al. [16] found that UBQLN4 is overexpressed in aggressive tumors and inhibits homologous recombination, redirecting double-strand break repair to nonhomologous end joining, resulting in increased genome instability and inducing carcinogenesis. Bustos et al. [17] found that the combination of UBQLN4 and ubiquitinated MRE11A induces the degradation of MRE11A by the proteasome, thereby regulating the level of the MRE11A protein after DNA damage and increasing the resistance of esophageal squamous cell carcinoma to cisplatin. However, the expression and function of UBQLN4 in CRC have not been reported. Here, we detected the expression, function and potential mechanisms of UBQLN4 in CRC.

In our study, C/EBPβ-activated UBQLN4 was upregulated in human CRC tissues and closely associated with the CRC size, TNM stage, and lymphatic metastasis. UBQLN4 activated the Wnt/β-catenin signaling pathway, thus promoting CRC proliferation and metastasis. Our study provides a novel insight into the mechanisms of CRC and a new biomarker for the diagnosis and management of CRC.

## Results

### UBQLN4 is upregulated in CRC and related to the CRC size, TNM stage, and lymphatic metastasis

**Fig. 1:**
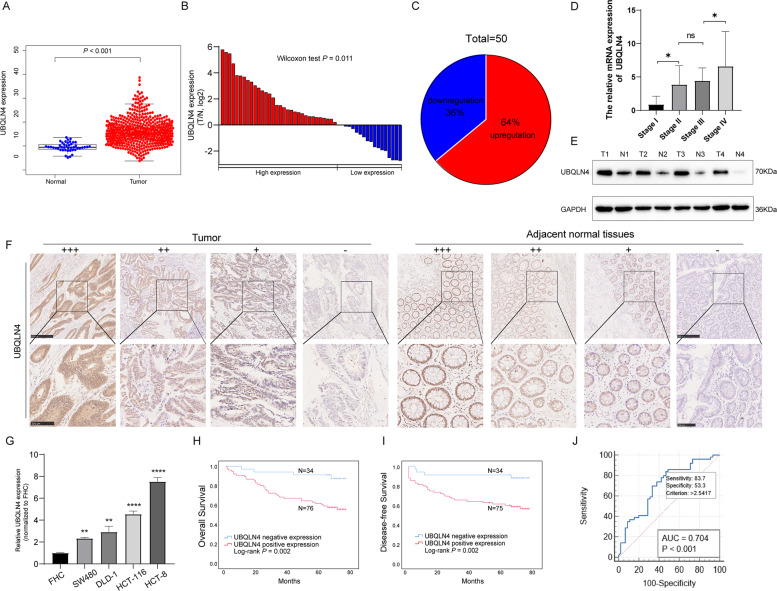
UBQLN4 is upregulated in CRC tissues and related to a poor prognosis. **A** TCGA database analysis showed significantly higher UBQLN4 expression in CRC tissues (*n* = 568) than in ANTs (*n* = 44) (*p* < 0.001). **B** The fold changes (log2) in UBQLN4 expression in 50 paired CRC tissues compared with ANTs detected using RT-qPCR. **C** Based on the fold changes in UBQLN4 expression, 50 paired CRC tissue samples were split into two groups. **D** The correlation between UBQLN4 expression and tumor stage. **E** UBQLN4 protein expression was examined in four paired CRC tissue samples using western blot. **F** Representative images of UBQLN4 IHC staining in CRC tissues and ANTs. Upper: 100×, scale bar: 250 μm. Lower: 200×, scale bar: 100 μm. **G** UBQLN4 expression was detected using RT-qPCR in FHC, SW480, DLD-1, HCT-116, and HCT-8 cells. **H**, **I** Kaplan–Meier OS and DFS curves showed that patients with higher UBQLN4 expression had shorter survival times. **J** ROC curves were used to estimate the diagnostic efficacy of UBQLN4 expression for CRC. All data from at least three independent experiments are presented as the means ± SD. *p < 0.05, **p < 0.01, *** or ****p < 0.001.

We detected the expression of UBQLN4 in CRC using The Cancer Genome Atlas (TCGA) database. UBQLN4 expression in CRC was higher than that in adjacent normal tissues (ANTs) (*p* < 0.001, Fig. 1A). We validated this result by measuring UBQLN4 expression in 50 paired CRC tissues and ANTs using real-time quantitative PCR (RT-qPCR) (Fig. 1B). Thirty-two patients (64%) showed higher expression of UBQLN4 in CRC tissues than in matched ANTs (Fig. 1C), and UBQLN4 expression increased as the tumor progressed (Fig. 1D). Western blot analysis of four pairs of CRC tissues also showed that UBQLN4 expression was increased in CRC (Fig. 1E). We also detected UBQLN4 expression in a retrospective cohort of 112 patients with CRC using immunohistochemistry (IHC) and observed higher UBQLN4 expression in CRC tissues than in ANTs; representative images of UBQLN4 IHC staining in CRC tissues and ANTs are shown in Fig. 1F. Moreover, UBQLN4 expression was higher in CRC cells than the human colonic epithelial cell line FHC (Fig. 1G).

Patients were grouped into a negative expression cohort and a positive expression cohort according to UBQLN4 IHC score of their CRC tissues to investigate the correlation between UBQLN4 and clinicopathological parameters (Table 1). UBQLN4-positive rates in CRC tissues and ANTs were 69.64% and 49.11%, respectively (*p* < 0.001). The correlations between UBQLN4 expression and clinicopathological characteristics were analyzed using the chi-square test. High UBQLN4 expression was positively correlated with the tumor size (*p* = 0.017), TNM stage (*p* = 0.038), and lymph node metastasis (N stage) (*p* = 0.020) (Table 2). Then, we evaluated the prognostic value of UBQLN4 in CRC patients using Kaplan–Meier analysis and the log-rank test, and patients with high UBQLN4 expression experienced shorter survival (overall survival, *p* = 0.002, Fig. 1H, disease-free survival, DFS, *p* = 0.002, Fig. 1I). The receiver operating characteristic curve indicated that UBQLN4 could be a potential diagnostic marker for CRC, with an area under the curve (AUC) greater than 0.7 (AUC = 0.704, *p* < 0.001, Fig. 1J). In addition, the multivariate analyses with the Cox regression model indicated that a positive N stage (*p* = 0.015), positive distant metastasis (*p* < 0.001) and high UBQLN4 expression (*p* = 0.024) were independent prognostic factors (Table 3).

**Table 1:** Expression level of UBQLN4 in colorectal tissue immunohistochemistry sections.

Histologic classification		UBQLN4 expression level				Percentage of positive samples (%)	*p* value
	*n*	(−)	(+)	(+ +)	(+ + +)		
Adjacent normal tissue	112	57	43	10	2	49.11	*p* < 0.001^a^
Colorectal cancer tissue	112	34	38	32	8	69.64	

*n* number.

^a^Statistical significance.

**Table 2:** Association between UBQLN4 expression and clinicopathological features in CRC patients (*n* = 112).

Variables	Patients (*n* = 112)	UBQLN4 expression level		*χ*^2^	*p* value
		Negative	Positive		
Gender				0.008	0.930
Male	60	18	42		
Female	52	16	36		
Age				0.046	0.831
≤60	51	16	35		
>60	61	18	43		
Tumor diameter (cm)				5.672	0.017^a^
≤5	86	31	55		
>5	26	3	23		
Tumor differentiation				0.507	0.477
Poor	28	7	21		
Well/Moderate	84	27	57		
TNM stage				4.300	0.038^a^
I–II	66	25	41		
III–IV	46	9	37		
T stage				0.931	0.335
T1-2	16	7	9		
T3-4	96	27	69		
N stage				5.398	0.020^a^
N0	67	27	44		
N1-2	45	7	34		
M stage				1.531	0.216
M0	95	31	64		
M1	17	3	14		

*CRC* colorectal cancer, *n* number.

^a^Statistical significance.

**Table 3:** Univariate and multivariate analysis of overall survival in CRC.

Variables	Univariate analysis		Multivariate analysis	
	HR (95% CI)	*p* value	HR (95% CI)	*p* value
Gender (male vs female)	1.20 (0.64–2.30)	0.550		
Age (≤60 years vs >60 years)	1.90 (0.95–3.80)	0.071		
Tumor diameter (≤5 cm vs >5 cm)	1.30 (0.65–2.80)	0.440		
Differentiation (well/moderate vs poor)	1.90 (0.94–3.80)	0.075		
TNM stage (I–II vs III–IV)	4.60 (2.30–9.30)	<0.001^a^	0.67 (0.18–2.51)	0.550
T stage (T1–T2 vs T3–T4)	1.30 (0.46–3.70)	0.620		
N stage (N0 vs N1–N2)	4.30 (2.20–8.50)	<0.001^a^	4.10 (1.30–13.00)	0.015^a^
M stage (M0 vs M1)	6.20 (3.10–12.0)	<0.001^a^	5.90 (2.50–14.00)	<0.001^a^
UBQLN4 expression (negative vs positive)	4.50 (1.60–13.0)	0.005^a^	3.40 (1.20–9.70)	0.024^a^

*CRC* colorectal cancer, *HR* hazard ratio, *CI* confidence interval.

^a^Statistical significance.

### UBQLN4 promotes CRC cell proliferation, migration, and invasion in vitro

**Fig. 2:**
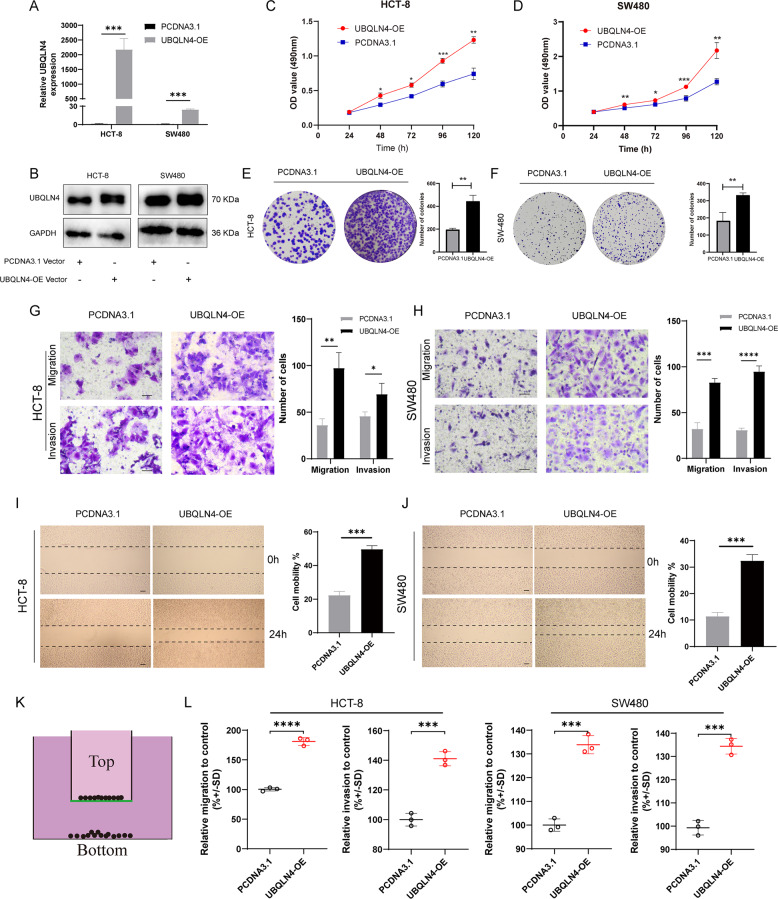
Upregulation of UBQLN4 promotes CRC cell proliferation, migration, and invasion in vitro. **A**, **B** Overexpression of UBQLN4 by transfecting a plasmid in both HCT-8 and SW480 cells. The efficiency was detected using RT-qPCR (**A**) and western blot (**B**). The MTS experiment indicated that UBQLN4 overexpression promoted the growth of both HCT-8 (**C**) and SW480 cells (**D**). The plate colony formation experiment was also utilized to detect the effect of UBQLN4 overexpression on the proliferation of HCT-8 (**E**) and SW480 cells (**F**). Transwell experiment of the migration and invasion of UBQLN4-overexpressing HCT-8 (**G**) and SW480 cells (**H**). 200×, Scale bar: 200 μm. Wound healing experiment showing the migration of UBQLN4-overexpressing HCT-8 (**I**) and SW480 cells (**J**). 100×, Scale bar: 200 μm. Schematic of the CytoSelect™ 24-Well Cell Migration Assay (**K**) and respective quantification of HCT-8 or SW480 cell migration and invasion after the upregulation of UBQLN4 (**L**). All data from at least three independent experiments are presented as the means ± SD. *p < 0.05, **p < 0.01, *** or ****p < 0.001.

**Fig. 3:**
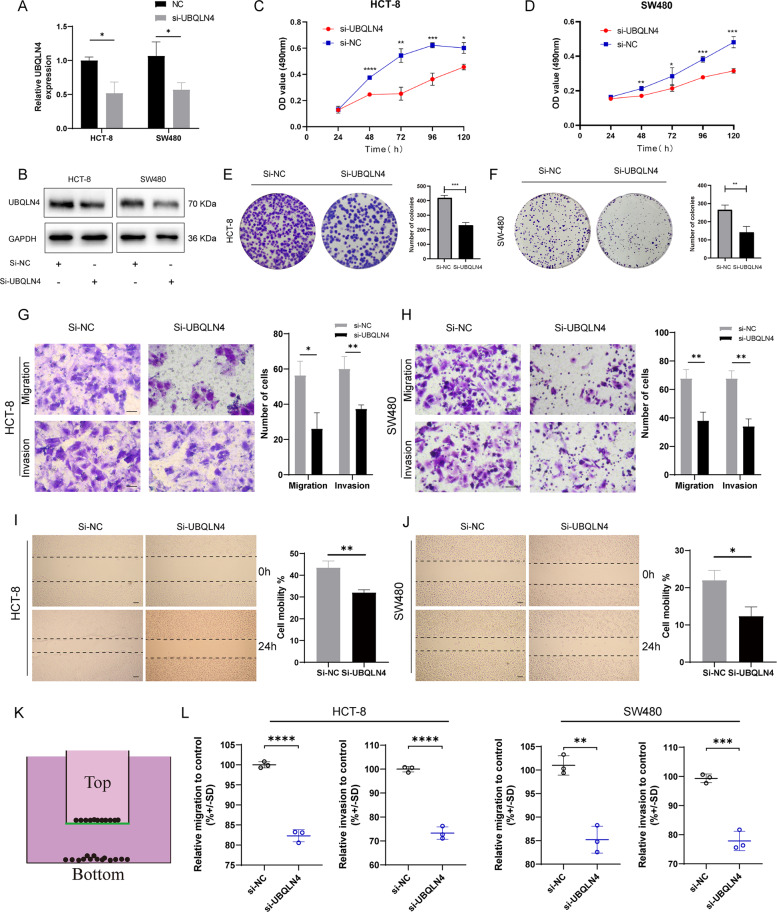
Downregulation of UBQLN4 inhibits CRC cell proliferation, migration, and invasion in vitro. **A**, **B** Knockdown of UBQLN4 by transfecting si-UBQLN4 in both HCT-8 and SW480 cells. The efficiency was detected using RT-qPCR (**A**) and western blot (**B**). The MTS experiment indicated that UBQLN4 knockdown inhibited the growth of both HCT-8 (**C**) and SW480 cells (**D**). The plate colony formation experiment was also used to detect the effect of UBQLN4 knockdown on the proliferation of HCT-8 (**E**) and SW480 cells (**F**). Transwell experiment of the migration and invasion of UBQLN4 knockdown HCT-8 (**G**) and SW480 cells (**H**). 200×, Scale bar: 200 μm. Wound healing experiment showing the effect of UBQLN4 knockdown on HCT-8 (**I**) and SW480 cell (**J**) migration. 100×, Scale bar: 200 μm. Schematic of the CytoSelect™ 24-Well Cell Migration Assay (**K**) and respective quantification of HCT-8 or SW480 cell migration and invasion after the downregulation of UBQLN4 (**L**). All data from at least three independent experiments are presented as the means ± SD. *p < 0.05, **p < 0.01, *** or ****p < 0.001.

We investigated the UBQLN4 biological effect by overexpressing and silencing UBQLN4 through the transfection of a UBQLN4 overexpression plasmid and siRNA against UBQLN4, respectively. Successful overexpression and silencing were proven by RT-qPCR and western blot experiment in both HCT-8 and SW480 cells (Figs. 2A, B and 3A, B). The growth curves measured using MTS experiments indicated that UBQLN4 overexpression significantly promoted the growth of both HCT-8 and SW480 cells (Fig. 2C, D), whereas UBQLN4 knockdown significantly inhibited the growth of both HCT-8 and SW480 cells (Fig. 3C, D). This result was consistent with the conclusion that UBQLN4 expression correlated with the tumor size. Meanwhile, the plate colony formation experiment revealed the same function of overexpressing and silencing UBQLN4, as presented in Figs. 2E, F and 3E, F. Transwell experiment was utilized to examine the effects of overexpressing and silencing UBQLN4 on the metastatic ability of CRC cells. UBQLN4 overexpression substantially increased CRC cells migration and invasion (Fig. 2G, H), while UBQLN4 knockdown remarkably decreased both the migration and invasion of CRC cells (Fig. 3G, H). The wound healing experiment also showed the same results as Transwell experiment (Figs. 2I, J and 3I, J). The CytoSelect™ 24-Well Cell Migration Assay was used to quantify cell migration and also showed the same results as Transwell assay (Figs. 2L and 3L).

### C/EBPβ induces UBQLN4 transcription in CRC cells

**Fig. 4:**
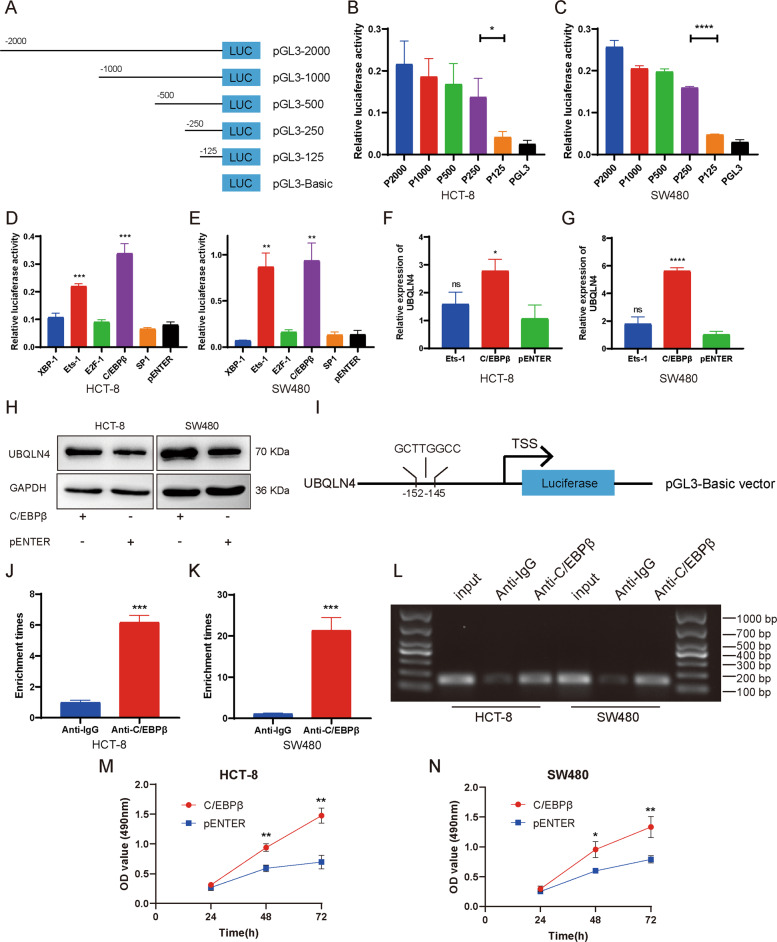
C/EBPβ induces UBQLN4 transcription in CRC cells. **A** Schematic illustration of pGL3-2000, pGL3-1000, pGL3-500, pGL3-250, pGL3-125, and pGL3-Basic. **B**, **C** Dual luciferase reporter assay was utilized to detect the core promoter region of UBQLN4. Overexpression of Ets-1 and C/EBPβ increased the luciferase activity of pGL3-250 in HCT-8 (**D**) and SW480 cells (**E**). RT-qPCR experiment showed that C/EBPβ upregulated UBQLN4 expression in HCT-8 (**F**) and SW480 cells (**G**). **H** Western blot experiment indicated that C/EBPβ increased UBQLN4 protein expression in both HCT-8 and SW480 cells. **I** Schematic diagram of the binding site for C/EBPβ in the UBQLN4 promoter region. **J**–**L** ChIP-qPCR showed greater enrichment of promoter amplicons of UBQLN4 in the anti-C/EBPβ group than in the IgG group of both HCT-8 and SW480 cells. The MTS experiment indicated that overexpression of C/EBPβ promoted the growth of both HCT-8 (**M**) and SW480 cells (**N**). All data from at least three independent experiments are presented as the means ± SD. ns not significant, *p < 0.05, **p < 0.01, *** or ****p < 0.001.

Almost all genes in eukaryotes are regulated by transcription factors (TFs) [18]. We identified the TF regulating UBQLN4 expression by obtaining the base sequence 2000 bp upstream of the UBQLN4 transcription start site (TSS) from NCBI (https://www.ncbi.nlm.nih.gov/gene). Then, five regions upstream of the UBQLN4 TSS were inserted into the pGL3-basic vector (named pGL3-2000, pGL3-1000, pGL3-500, pGL3-250, and pGL3-125) (Fig. 4A). Dual luciferase reporter assay indicated that the relative luciferase activity of both HCT-8 and SW480 cells decreased remarkably between pGL3-250 and pGL3-125, indicating that this region is the core promoter region of UBQLN4 (Fig. 4B, C). The core promoter region of UBQLN4 from 125 to 250 bp upstream was analyzed by the PROMO 3.0 program (http://alggen.lsi.upc.es/cgi-bin/promo_v3/promo/promoinit.cgi?dirDB=TF_8.3). XBP-1, Ets-1, E2F-1, C/EBPβ, and SP1 had high scores and were identified as potential TFs binding to the core promoter region. Overexpression of Ets-1 and C/EBPβ substantially increased the luciferase activity of pGL3-250 in both HCT-8 and SW480 cells (Fig. 4D, E). We further clarified the TFs regulating UBQLN4 expression by performing RT-qPCR and western blot experiments. RT-qPCR experiment indicated that C/EBPβ overexpression significantly increased UBQLN4 expression in both HCT-8 and SW480 cells, while Ets-1 had no effect on UBQLN4 expression (Fig. 4F, G). Western blot experiment also showed that C/EBPβ induced UBQLN4 expression in both HCT-8 and SW480 cells compared with the negative control (NC) group (Fig. 4H). Then, we verified the direct interaction between C/EBPβ and the UBQLN4 core promoter region (Fig. 4I) by performing chromatin immunoprecipitation (ChIP) experiment. In both HCT-8 and SW480 cells, compared with the NC group (anti-IgG), anti-C/EBPβ significantly enriched the chromatin containing the binding site corresponding to C/EBPβ (Fig. 4J, K). As shown in Fig. 4L, PCR products were amplified from DNA fragments immunoprecipitated with the anti-C/EBPβ antibody using primers covering the binding site of C/EBPβ. Meanwhile, the growth curves measured using MTS experiment indicated that C/EBPβ overexpression significantly induced the growth of both HCT-8 and SW480 cells (Fig. 4M, N).

### UBQLN4 exerts oncogenic effects by activating the Wnt/β-catenin signaling pathway

**Fig. 5:**
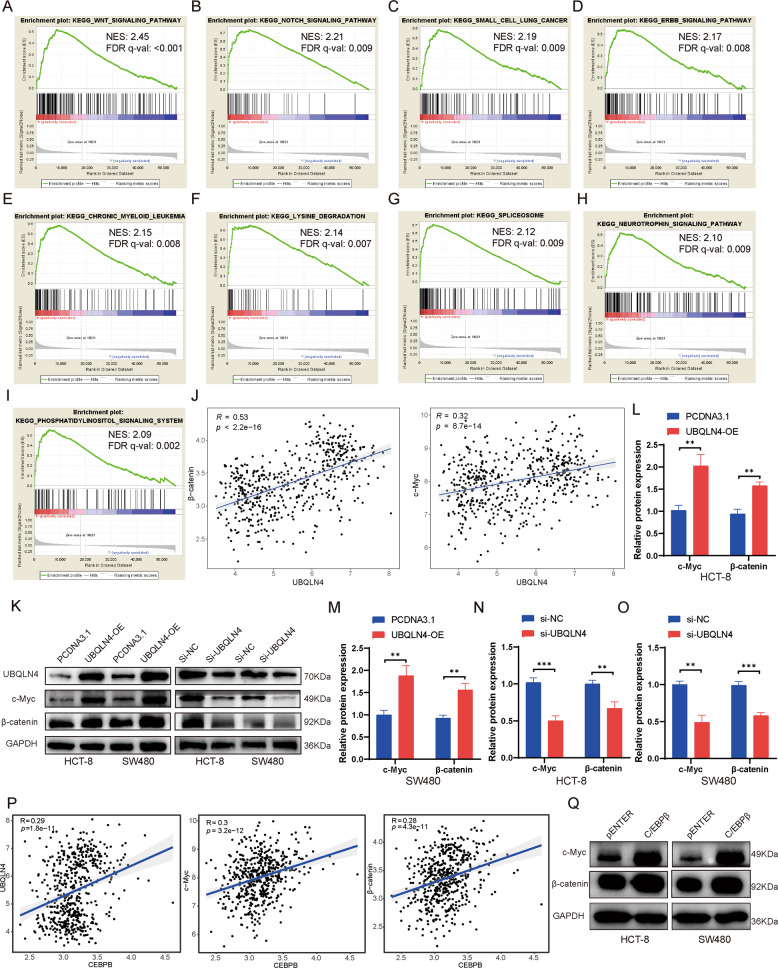
UBQLN4 exerts oncogenic effects by activating the Wnt/β-catenin signaling pathway. **A**–**I** The top 9 pathways enriched in the high UBQLN4 expression phenotype (sorted by NES). **J** The correlations between the expression of UBQLN4 and β-catenin and UBQLN4 and c-Myc (Data were obtained from GEO database, GSE131418). **K**–**O** Western blot experiment indicated that UBQLN4 overexpression increased the expression of β-catenin and c-Myc, while UBQLN4 knockdown reduced the expression of β-catenin and c-Myc. **P** The correlation levels between C/EBPβ and UBQLN4, C/EBPβ and c-Myc, and C/EBPβ and β-catenin (Data were obtained from GEO database, GSE131418). **Q** Western blot showed that C/EBPβ overexpression increased β-catenin and c-Myc levels in both HCT-8 and SW480 cells. All data from at least three independent experiments are presented as the means ± SD. *p < 0.05, **p < 0.01, *** or ****p < 0.001.

We performed a gene set enrichment analysis (GSEA) on the transcriptome data from all CRC patients in TCGA database to explore the molecular mechanism underlying the cancer-promoting effect of UBQLN4. Patients were grouped into a UBQLN4 high expression group and a UBQLN4 low expression group as per the median UBQLN4 expression level. Based on the normalized enrichment score (NES), we selected the top 9 gene sets with the highest scores as described below (Fig. 5A–I). GSEA indicated that the NES of the Wnt/β-catenin signaling pathway was 2.45 and the false discovery rate (FDR) *q* value <0.001, indicating that UBQLN4 is likely to exert its cancer-promoting effect via activating the Wnt/β-catenin signaling pathway. We verified this hypothesis by analyzing the GEO database (GSE131418) to explore the relationship between the expression of UBQLN4 and β-catenin, UBQLN4 and c-Myc, an important target gene downstream of the Wnt/β-catenin signaling pathway. UBQLN4 expression was remarkably positively correlated with β-catenin and c-Myc expression (Fig. 5J). Western blot experiment indicated that UBQLN4 overexpression remarkably increased β-catenin and c-Myc expression in both HCT-8 and SW480 cells, while UBQLN4 knockdown significantly reduced β-catenin and c-Myc expression in both HCT-8 and SW480 cells (Fig. 5K–O). Then, we explored whether C/EBPβ regulated the Wnt/β-catenin signaling pathway by analyzing the correlation between C/EBPβ and UBQLN4, C/EBPβ and c-Myc, and C/EBPβ and β-catenin using the GEO database (GSE131418). The results are shown in Fig. 5P. We found that C/EBPβ expression was significantly positively correlated with the expression of UBQLN4 (*R* = 0.29, *p* < 0.001), c-Myc (*R* = 0.30, *p* < 0.001), and β-catenin (*R* = 0.28, *p* < 0.001). Western blot experiment also indicated that C/EBPβ overexpression promoted β-catenin and c-Myc expression in both HCT-8 and SW480 cells (Fig. 5Q). Based on these experiments, UBQLN4 is activated by C/EBPβ and exerts its oncogenic effects on CRC by activating the Wnt/β-catenin signaling pathway to upregulate β-catenin expression, which further activates the transcription of the downstream target gene c-Myc and upregulates c-Myc expression.

### UBQLN4-mediated effects are abolished by the downregulation of c-Myc

**Fig. 6:**
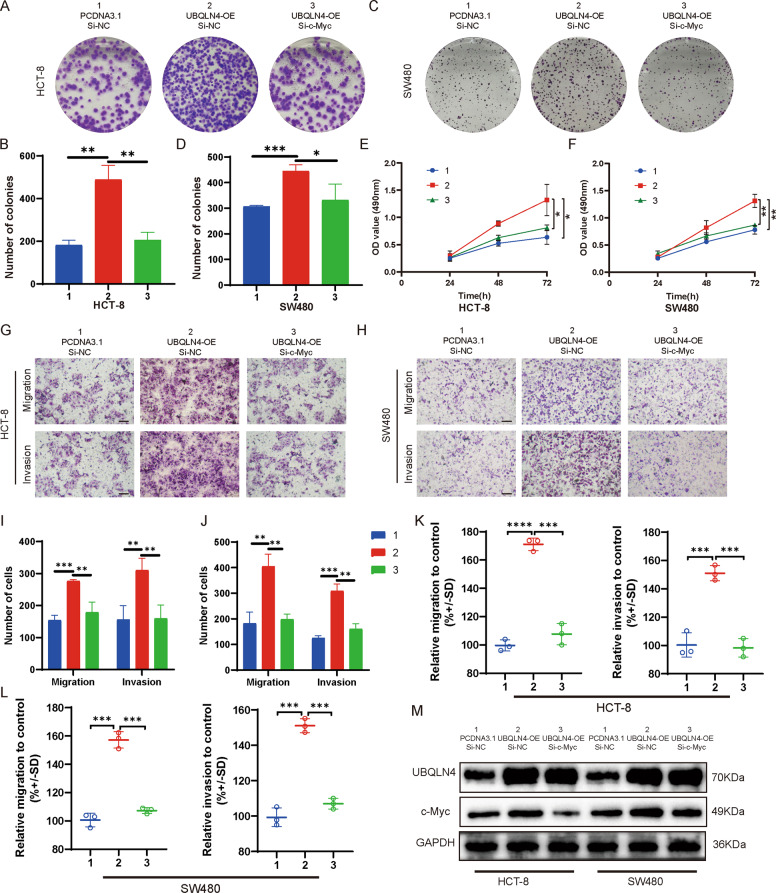
UBQLN4-mediated effects are abolished by the downregulation of c-Myc. Plate colony formation experiment showed that silencing c-Myc expression in both HCT-8 (**A**, **B**) and SW480 cells (**C**, **D**) reversed the increased proliferation induced by UBQLN4 overexpression. The MTS experiment also showed that silencing c-Myc expression in both HCT-8 (**E**) and SW480 cells (**F**) reversed the increased proliferation induced by UBQLN4 overexpression. **G**–**L** Transwell and CytoSelect™ 24-Well Cell Migration assays indicated that silencing c-Myc expression in both HCT-8 and SW480 cells abolished the increased migration and invasion induced by upregulation of UBQLN4, 100×, Scale bar: 400 μm. **M** Western blot showed that silencing c-Myc expression in both HCT-8 and SW480 cells abolished the increased expression of c-Myc induced by the upregulation of UBQLN4. All data from at least three independent experiments are presented as the means ± SD. *p < 0.05, **p < 0.01, *** or ****p < 0.001.

Considering that UBQLN4 activates the Wnt/β-catenin signaling pathway, we further explored whether c-Myc mediates the effects of UBQLN4 on promoting CRC cell proliferation, migration, and invasion. We confirmed this hypothesis by conducting a rescue experiment to verify that silencing c-Myc expression abolished the CRC cells malignant phenotype induced by upregulated the expression of UBQLN4. The results of the plate colony formation experiment indicated that silencing c-Myc expression in both HCT-8 and SW480 cells significantly reversed the increased proliferation induced by UBQLN4 overexpression (Fig. 6A–D). The MTS experiment also produced the same results as the plate colony formation experiment (Fig. 6E, F). Meanwhile, Transwell and CytoSelect™ 24-Well Cell Migration assays showed that silencing c-Myc expression in both HCT-8 and SW480 cells significantly abolished the increased migration and invasion induced by upregulating UBQLN4 expression (Fig. 6G–L). Western blot analysis indicated that silencing c-Myc expression in both HCT-8 and SW480 cells abolished increased c-Myc expression induced by the upregulation of UBQLN4 (Fig. 6M).

### Downregulation of UBQLN4 inhibits CRC growth in vivo

**Fig. 7:**
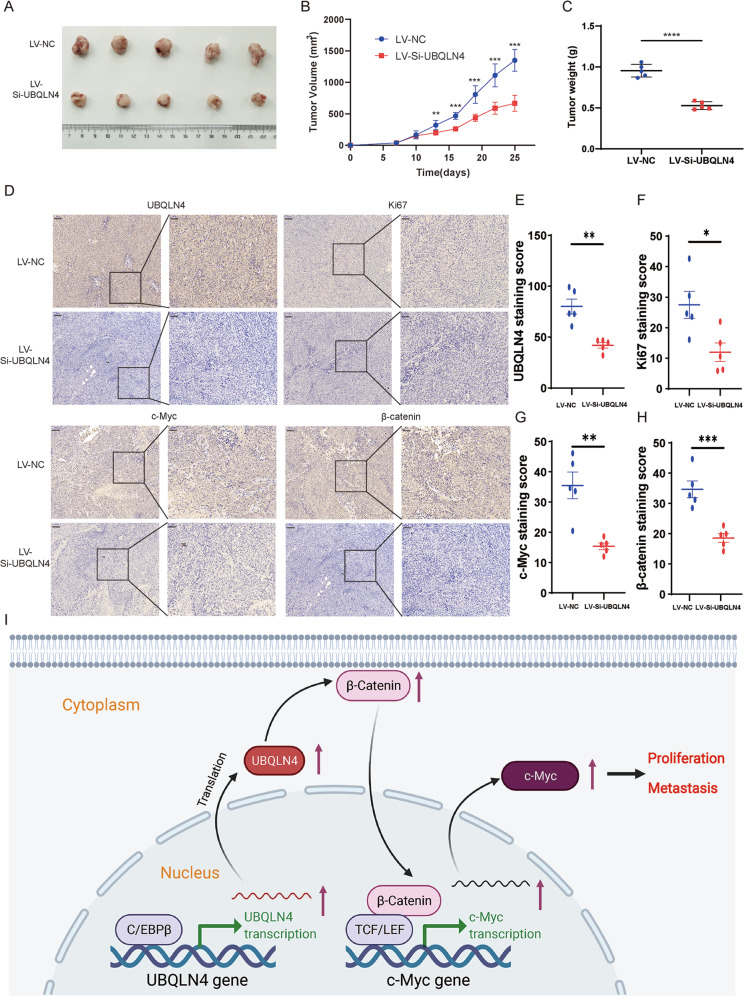
Downregulation of UBQLN4 inhibits CRC growth in vivo. **A** The size of xenograft tumors after harvest. **B** The growth curve of xenograft tumors from tumor formation to sacrifice in mice. **C** The weight of xenograft tumors after harvest. **D**–**H** IHC staining of xenograft tumors indicated that the LV-Si-UBQLN4 group showed lower UBQLN4, β-catenin, c-Myc and Ki67 expression than the LV-NC group. Left: 100×, scale bar: 100 μm. Right: 200×, scale bar: 50 μm. **I** Schematic illustration of the UBQLN4 molecular mechanism in CRC. All data from at least three independent experiments are presented as the means ± SD. *p < 0.05, **p < 0.01, *** or ****p < 0.001.

An in vivo experiment was performed to investigate the function of UBQLN4 in driving tumorigenesis. First, xenografts were established by subcutaneous injection of SW480 cells into the right flank of BALB/c nude mice. Then, we purchased the UBQLN4 knockdown lentivirus (LV-Si-UBQLN4) and NC lentivirus (LV-Si-UBQLN4) and injected them into the tumors. Compared to the LV-NC group, the LV-Si-UBQLN4 group had a much smaller mean tumor volume and tumor weight (Fig. 7A–C), manifesting that targeted knockdown of UBQLN4 expression effectively inhibited tumor proliferation in vivo. IHC staining in xenograft tumors verified that the LV-Si-UBQLN4 group exhibited lower UBQLN4, β-catenin, c-Myc and Ki67 expression than the LV-NC group (Fig. 7D–H). These collective results showed that UBQLN4 plays a vital role in promoting CRC proliferation, migration, and invasion through the Wnt/β-catenin signaling pathway.

## Discussion

Numerous studies have shown that alterations in ubiquitination signaling pathways are present in a various of human health-threatening diseases, such as malignancies and neurodegenerative diseases [19–22]. Since the ubiquitin–proteasome system plays an important regulatory role in numerous diseases, many proteins with a prominent role in this system have become popular targets for current drug design and discovery. Regulation of aberrant ubiquitination networks in diseases by developing inhibitors that specifically interfere with the degradation of substrate proteins achieves targeted and precise treatment of malignant diseases [23]. UBQLN4 is an important regulator of the ubiquitin–proteasome system. Our study confirmed that UBQLN4 is upregulated in CRC. High expression of UBQLN4 is positively associated with the CRC size, TNM stage and lymph node metastasis (N stage), and patients with high UBQLN4 expression have a poor prognosis. MTS and plate colony formation experiments showed that UBQLN4 increased CRC cell proliferation, consistent with the result that UBQLN4 expression correlated with tumor size. Transwell and wound healing experiments manifested that UBQLN4 increased CRC cells migration and invasion, consistent with the result that UBQLN4 expression associated with lymph node metastasis.

UBQLN4 was clearly identified as a key molecule regulating the proliferation and metastasis of CRC. However, the regulatory factors involved in aberrant UBQLN4 expression in CRC remain unclear. Parbin et al. [24] found that low expression of microtubule-associated tumor suppressor 1 (MTUS1) is associated with DNA methylation and histone deacetylation in human non-small cell lung cancer. Baylin et al. [25] illustrated that histone hyperacetylation is usually present in the promoter region of proto-oncogenes in tumor tissues, which may induce the expression of oncogenes. According to Skipper et al. [18], almost all genes in eukaryotes are regulated by TFs. As shown in the study by Fang et al. [26], they confirmed that Ets-1 directly binds to the CCR7 promoter and induces the transcription of CCR7, which upregulates CCR7 expression. Therefore, we considered whether UBQLN4 is also regulated by TFs, leading to its abnormally high expression in CRC. C/EBPβ is a member of the family of TFs containing the C/EBP leucine zipper domain [27, 28]. Similar to other C/EBP family proteins, this intron-free gene product binds to certain genomic regulatory regions in the form of homodimers or heterodimers with other molecules to induce or inhibit target genes expression. C/EBPβ plays a vital role in regulating histone methylation, promoter methylation, cell senescence and glycolysis [28–32]. C/EBPβ, a TF regulating the expression of the lncRNA UCA1, upregulates lncRNA UCA1 expression and subsequently promotes bladder cancer proliferation [33]. In our study, we confirmed that C/EBPβ directly bound to the core promoter region of UBQLN4 and induced the transcription of UBQLN4, which upregulated UBQLN4 expression in CRC.

The Wnt/β-catenin signaling cascade is a major regulator of development throughout the animal kingdom [34]. Mutations in the components of the Wnt/β-catenin pathway lead to a variety of growth- and development-related diseases and cancers [35–37]. Previous studies have identified mutations in Wnt/β-catenin pathway-related components in several tumors [38, 39]. Korinek et al. [40] first discovered the mutation of APC, a key gene in the Wnt/β-catenin signaling pathway, in familial adenomatous polyposis in 1997. According to Kinzler et al. [41], most cases of sporadic CRC are due to the loss of two APC alleles. Rubinfeld et al. [42] also identified β-catenin mutations in melanoma. In addition, similar β-catenin mutations have been observed in many other cancers [43, 44]. Meanwhile, c-Myc, a crucial target gene downstream of the Wnt/β-catenin signaling pathway, promotes the development and progression of many tumors [45]. Based on these studies, abnormalities in the Wnt/β-catenin signaling pathway are closely related to tumorigenesis and progression. In our study, we found that UBQLN4 expression was positively correlated with β-catenin and c-Myc expression by analyzing the GEO database (GSE131418). Western blot experiments further validated this result, indicating that UBQLN4 upregulated β-catenin and c-Myc expression.

In summary, our study identified UBQLN4 as an independent prognostic factor for CRC patients, and patients with high UBQLN4 expression had a poor prognosis. UBQLN4 expression induced by the TF C/EBPβ promoted CRC proliferation, migration, and invasion. In addition, UBQLN4 activated the Wnt/β-catenin signaling pathway to upregulate β-catenin and c-Myc expression, thereby promoting CRC proliferation, migration, and invasion (Fig. 7I). These findings indicate the importance of UBQLN4 in the development and progression of CRC, and UBQLN4 may be a promising target for the diagnosis and treatment of patients with CRC.

## Materials and methods

### Tissue samples

All CRC and ANTs were obtained from 112 CRC patients who underwent radical resection of CRC between 2015 and 2018 at the Department of Gastrointestinal Surgery, Qilu Hospital of Shandong University, China. All tissues were divided into two parts: one part was frozen in liquid nitrogen at −80 °C immediately after isolation, and the other part was fixed with 10% neutral formalin tissue fixative and embedded in paraffin before sectioning for subsequent experiments. The inclusion and exclusion criteria were consistent with our previous study [46]. This study protocol was approved by the Ethics Committee of Qilu Hospital of Shandong University. All patients provided their signed informed consent.

### Cell lines

Four human CRC cell lines (HCT-8, SW480, DLD-1, and HCT-116) and a normal human colonic epithelial cell line (FHC) were purchased from the Cell Bank of the Chinese Academy of Science (Shanghai, China). HCT-116 and SW480 cell lines were maintained in Dulbecco’s modified Eagle’s medium (DMEM, Gibco, Grand Island, NY, USA) supplemented with 10% fetal bovine serum (FBS, Wisent, Montreal, Canada). HCT-8 and DLD-1 cell lines were cultivated in RPMI-1640 medium (HyClone, Logan, Utah, USA) supplemented with 10% FBS. FHC was maintained in Ham’s F12 nutrient medium (F12, Gibco, Grand Island, NY, USA) supplemented with 10% FBS. Cells were cultured in a 37 °C incubator containing 5% CO_2_. All cells were identified by short tandem repeat DNA fingerprinting. Cultures were regularly assessed for mycoplasma. No mycoplasma was detected in any cell lines when the experiments were in progress.

### RNA extraction and real-time quantitative PCR experiments

Total RNA was extracted with TRIzol reagent (Invitrogen, Carlsbad, CA, USA) from tissues or cells as per the manufacturer’s protocol. Then, 1 μg of total RNA was reverse transcribed to cDNAs via the PrimeScript^TM^ RT Reagent Kit (Takara, Tokyo, Japan). Real-time PCR was implemented via SYBR Premix Ex Taq (Takara, Tokyo, Japan). The results were normalized to GAPDH expression and calculated via the 2^−ΔΔCT^ method. All primer sequences are displayed in Supplementary Table 1.

### Western blot experiment

Total proteins were extracted with RIPA cell lysis buffer (Beyotime, Shanghai, China) from tissues or cells as per the manufacturer’s protocol. Extracted proteins were electrophoretically separated and transferred to PVDF membranes (Pierce Biotechnology, USA). PVDF membranes were blocked with PBS buffer containing 5% skim milk power and maintained in primary antibodies overnight at 4 °C. Secondary antibodies (1:5000) were incubated with the membrane for 1 h. The primary antibodies included UBQLN4 (1:1000 dilution, Santa Cruz, sc-136145), GAPDH (1:1000 dilution, CST, # 5174S), c-Myc (1:1000 dilution, CST, # 9402S), and β-catenin (1:1000 dilution, CST, # 8480S).

### Immunohistochemistry

IHC was performed as previously described [47]. The IHC score was independently calculated by two professional pathologists. The intensity of cellular staining was divided into four levels: 0: negative staining; +1: weak staining; +2: moderate staining; and +3: strong staining. The area of positive cells was assessed and recorded as a percentage: 0: (no positive cells); +1: <10%; +2: 10% to 50%; +3: >50%. The two scores were multiplied to produce the final score: negative cases (−, final score ≤ 1), weak positive cases (+, final score: 2–3), moderate positive cases (++, final score: 4–6), and strong positive cases (+++, 7–9). Antibodies against UBQLN4 (1:50 dilution, Santa, sc-136145), c-Myc (1:1000 dilution, CST, # 9402S), and β-catenin (1:1000 dilution, CST, # 8480S) and Ki67 (1:200 dilution, Invitrogen, PA5-114437) were used.

### Cell transfection and vector construction

The siRNAs, NC, UBQLN4 overexpression plasmid and pcDNA3.1 (+) vector were purchased from GenePharma (Shanghai, China). We overexpressed or knocked down the expression levels of proteins by transfecting gene-specific overexpression plasmids or siRNAs into cells via Lipofectamine 2000 (Thermo Fisher Scientific, MA, USA). The siRNA sequences are displayed in Supplementary Table 1.

### MTS and plate colony formation experiments

The proliferation of CRC cells was assessed using the MTS reagent (Abcam, ab197010, USA) and plate colony formation experiments. For the MTS experiment, 1000 CRC cells transfected with si-NC, si-UBQLN4, pcDNA3.1, or the UBQLN4 overexpression plasmid were seeded into each well of 96-well plates with 100 μl of complete DMEM or RPMI-1640 medium. At 0, 24, 48, 72, 96, and 120 h after transfection, 20 μl of MTS reagent were added to each well and then incubation was continued for 2 h at 37 °C. The absorbance was measured at 490 nm. For the plate colony formation experiment, 1000 CRC cells transfected with si-NC, si-UBQLN4, pcDNA3.1, or the UBQLN4 overexpression plasmid were seeded in six-well plates, and the number of clones was enumerated 7 days after plating.

### Transwell and wound healing experiments

Transwell migration experiments were performed via Transwell inserts (Costar, NY, USA) with 8 μm pores. Cells were transfected with si-NC, si-UBQLN4, pcDNA3.1, or UBQLN4 overexpression plasmids. HCT-8 cells (7 × 10^4^) and SW480 cells (1 × 10^5^, gain of function) were seeded in the upper chambers in serum-free medium, and complete medium (0.6 ml) was added to the lower chamber. After an incubation for 48 h at 37 °C, the cells that had migrated to the lower surface of the upper chambers were fixed with methanol, stained with crystal violet and imaged with a microscope at ×100 (×200) magnification. When we performed a loss-of-function experiment, 1 × 10^5^ HCT-8 cells and 2 × 10^5^ SW480 cells were plated in the upper chambers. When we performed a cell invasion experiment, all steps were the same as those described above, except that the upper chamber was coated with Matrigel (BD Biosciences, New Jersey, USA). For the quantitative determination of cell migration, the Transwell migration assay was also conducted with the “CytoSelect™ 24-well cell migration assay kit 8 µm” (Cell Biolabs Inc.) as per the manufacturer’s instructions. For wound healing experiments, we performed the experiments using previously described methods [48].

### In vivo assay

The animal experiment was performed with approval from the Medical Ethics Committee of Shandong University. Ten male BALB/c nude mice (3–4 weeks old) were purchased from Charles River Biotechnology (Beijing, China). Mice were split into two groups randomly (*n* = 5 mice per group). The SW480 cell suspension (2 × 10^6^ cells/0.1 ml of PBS) was inoculated subcutaneously in the right anterior axilla of each mouse. LV-Si-UBQLN4 and LV-NC were injected into the tumor every 3 days after tumors formed, and the volume of the tumor was measured at the same time. After 26 days, the mice were sacrificed. Tumors were excised and weighed, and immunohistochemical staining was performed. The formula for calculating tumor volume (*V*) was *V* = (long diameter) × (short diameter)^2^/2. All subcutaneous tumors did not exceed 2.0 cm in diameter, and all mice appeared of good health throughout the experiment.

### Dual luciferase reporter assay

We obtained the base sequence 2000 bp upstream of the UBQLN4 TSS from NCBI (https://www.ncbi.nlm.nih.gov/gene). Then, five regions upstream of the UBQLN4 TSS were inserted into the pGL3-basic vector (named pGL3-2000, pGL3-1000, pGL3-500, pGL3-250, and pGL3-125) by BioSune (Jinan, China). XBP-1, Ets-1, E2F-1, C/EBPβ, and SP1 overexpression plasmids were purchased from GenePharma (Shanghai, China). We performed a dual luciferase reporter assay using a previously reported protocol [46].

### Chromatin immunoprecipitation assay

ChIP assays were performed in both HCT-8 and SW480 cells as previously described [49]. The anti-C/EBPβ (Proteintech, 23431-1-AP) and anti-IgG antibodies were used. Primers used in this experiment are displayed in Supplementary Table 1.

### Gene set enrichment analysis (GSEA)

In our study, we performed a GSEA on the transcriptome data from all CRC samples in TCGA database to explore the signaling pathways enriched in response to UBQLN4 overexpression. As per the median value of UBQLN4 expression, the samples were grouped into a UBQLN4 high expression group and a UBQLN4 low expression group. Gene set permutations were performed 1000 times per analysis. UBQLN4 expression level was used as a phenotypic label. Pathways enriched in each phenotype were sorted according to the nominal *p* value, NES, and FDR.

### Statistical analysis

GraphPad Prism 8.0 and R software (3.6.1) were used for statistical analyses and the visualization of all data in this study. Two-tailed Student’s *t* test, Wilcoxon test, and one-way analysis of variance were performed for statistical comparisons, as appropriate. The chi-square test was utilized to analyze the relationship between UBQLN4 expression and clinicopathological characteristics. Univariate and multivariate Cox proportional hazards regression models were used to analyze the prognostic significance of UBQLN4 expression in CRC patients. The Kaplan–Meier method and log-rank test were utilized to analyze the differences in the OS and DFS of patients with CRC presenting different UBQLN4 expression levels. All values are presented as the means ± standard deviations of three independent experiments. *p* < 0.05 was defined as statistically significant.

## Supplementary information

Supplementary Table 1

## Data Availability

The data in this work are available with the approval of corresponding authors in reasonable requirement.
