# Supplementary material for: UBQLN4 is activated by C/EBPβ and exerts oncogenic effects on colorectal cancer via the Wnt/β-catenin signaling pathway
Source: Cell Death Discov. 2021 Dec 20;7:398. doi: 10.1038/s41420-021-00795-4 (PMC8688525; doi:10.1038/s41420-021-00795-4)
Supplement: Supplementary file 1 — Supplementary Table 1 [file 41420_2021_795_MOESM1_ESM.docx]

**Supplementary Table 1. Primers and Oligonucleotides sequences**

| Variable | Sequence (5'-3') |
| --- | --- |
| Primers |  |
| UBQLN4 Forward | CGCATCAACGTGGTTGTAAA |
| UBQLN4 Reverse | ACCGTCAGGTTGTCCTTGAT |
| GAPDH Forward | CCACTGCCGAGGAGGTCAACTAC |
| GAPDH Reverse | ATGCTCAGGGTGATTGCGTATGC |
| ChIP-C/EBPβ Forward | CTCCGGACCTGGGGCTT |
| ChIP-C/EBPβReverse | TCCCCGAGGCTTCACCG |
| c-Myc Forward | ACAGCCCACTGGTCCTCAAG |
| c-Myc Reverse | TCGGTTGTTGCTGATCTGTCTC |
| siRNAs |  |
| si-NC | UUCUCCGAACGUGUCACGU |
|  | ACGUGACACGUUCGGAGAA |
| si-UBQLN4 | GGUCAGGGAUGUUCAAUAG |
|  | CAAUAACCCUGAACUCAUG |
| si-NC | CACCGAUAGUAGGUGCCGG |
|  | AAAAUAGUAGGUGCCGGAA |
| si-c-Myc | CGAUGUUGUUUCUGUGGAA |
|  | CCAAGGUAGUUAUCCUUAA |

**Abbreviations:** UBQLN4, ubiquilin 4; GAPDH, glyceraldehyde 3-phosphate dehydrogenase; ChIP, chromatin immunoprecipitation; NC, negative control.
